# AFM-YOLOv8s: An Accurate, Fast, and Highly Robust Model for Detection of Sporangia of *Plasmopara viticola* with Various Morphological Variants

**DOI:** 10.34133/plantphenomics.0246

**Published:** 2024-09-11

**Authors:** Changqing Yan, Zeyun Liang, Ling Yin, Shumei Wei, Qi Tian, Ying Li, Han Cheng, Jindong Liu, Qiang Yu, Gang Zhao, Junjie Qu

**Affiliations:** ^1^ College of Intelligent Equipment, Shandong University of Science and Technology, Taian 271019, China.; ^2^ Guangxi Crop Genetic Improvement and Biotechnology Key Lab, Guangxi Academy of Agricultural Sciences, Nanning 530007, China.; ^3^ College of Natural Resources and Environment, Northwest A&F University, Yangling, Shaanxi 712100, China.; ^4^ State Key Laboratory of Soil Erosion and Dryland Farming on the Loess Plateau, Northwest A&F University, Yangling 712100, China.; ^5^ BASF Digital Farming GmbH, Im Zollhafen 24, 50678 Köln, Germany.

## Abstract

Monitoring spores is crucial for predicting and preventing fungal- or oomycete-induced diseases like grapevine downy mildew. However, manual spore or sporangium detection using microscopes is time-consuming and labor-intensive, often resulting in low accuracy and slow processing speed. Emerging deep learning models like YOLOv8 aim to rapidly detect objects accurately but struggle with efficiency and accuracy when identifying various sporangia formations amidst complex backgrounds. To address these challenges, we developed an enhanced YOLOv8s, namely, AFM-YOLOv8s, by introducing an Adaptive Cross Fusion module, a lightweight feature extraction module FasterCSP (Faster Cross-Stage Partial Module), and a novel loss function MPDIoU (Minimum Point Distance Intersection over Union). AFM-YOLOv8s replaces the C2f module with FasterCSP, a more efficient feature extraction module, to reduce model parameter size and overall depth. In addition, we developed and integrated an Adaptive Cross Fusion Feature Pyramid Network to enhance the fusion of multiscale features within the YOLOv8 architecture. Last, we utilized the MPDIoU loss function to improve AFM-YOLOv8s’ ability to locate bounding boxes and learn object spatial localization. Experimental results demonstrated AFM-YOLOv8s’ effectiveness, achieving 91.3% accuracy (mean average precision at 50% IoU) on our custom grapevine downy mildew sporangium dataset—a notable improvement of 2.7% over the original YOLOv8 algorithm. FasterCSP reduced model complexity and size, enhanced deployment versatility, and improved real-time detection, chosen over C2f for easier integration despite minor accuracy trade-off. Currently, the AFM-YOLOv8s model is running as a backend algorithm in an open web application, providing valuable technical support for downy mildew prevention and control efforts and fungicide resistance studies.

## Introduction

Fungal and oomycete diseases pose a substantial threat to staple food crops, fruits, and vegetables, resulting in considerable economic losses [1,2]. Notably, in the case of oomycete diseases like grapevine downy mildew, the release and dispersion of sporangia are critical steps in the epidemiology of plant diseases [3]. Effective monitoring of spore dissemination—whether it is spores or sporangia (referred to as spores for simplicity)—using spore traps and microscopes, is essential for implementing timely intervention strategies and ensuring the quality and yield of the harvest [4,5]. In addition, evaluating the effectiveness of fungicides against various fungal strains requires regular observation of sporangia under a microscope. Manual identification and quantification of sporangia are hindered by their labor-intensive nature, restricted throughput, susceptibility to human error, and subjectivity, all of which compromise the reliability of the data [6,7]. Observer variability and fatigue worsen these issues, highlighting the necessity for automated, scalable approaches to enhancing disease detection accuracy and speed in agriculture.

To streamline spore monitoring and reduce manual efforts, machine learning techniques are increasingly used for automatic spore identification. For example, Lei et al. [5] used *K*-means clustering algorithm for detecting and quantifying urediniospores [8]. However, these segmentation-centric methods prove weakness for dense or occluded scenarios. Alternatively, Wang et al. [9] used a support vector machine for classification subsequent to extracting 13 features from diffraction fingerprint images of fungal spores and achieved a classification accuracy of 93.6%. While image processing and machine learning algorithms have shown good performances in spore detection, they are limited to single-target identification with distinct features and simple backgrounds. In scenarios involving multiple targets and complex backgrounds, traditional machine learning methods encounter challenges and are not practical yet for real-world applications [10–12].

In recent years, the advancements in deep neural network and graphic processing units have substantially increased accuracy and adoption of deep learning techniques for spore classification and detection. For instance, Crespo-Michel et al. [13] devised a novel classification method using ResNet50, VGG-16, MobileNet, and InceptionV3 networks, achieving an impressive 97.4% accuracy on a dataset of *Lasiodiplodia* fungal spores. Later, the introduction of You Only Look Once (YOLO) family of models offers advantages by directly predicting the entire image without generating candidate regions, maintaining high accuracy while reducing computational complexity and inference time [14]. The performance of the YOLO model has been consistently enhanced, with the state-of-the-art version, YOLOv8 [15]. In the field of utilizing and improving YOLO models for spore detection, Li et al. [6] enhanced spore detection capabilities by augmenting the YOLOv5 algorithm with multihead self-attention (MHSA) attention mechanism and GHOST lightweight module, achieving satisfactory performance in cucumber gray mold spore detection. However, its limitation lies in detecting only a single gray mold spore category. To address this, Zhao et al. [16] enhanced the ability of YOLOv8 to detect global information by incorporating the efficient channel attention (ECA) attention mechanism, enabling the model to detect and distinguish various morphologically similar spores. However, challenges remain when detecting objects undergoing substantial morphological changes and occlusions under complex environmental conditions [17,18].

The objective of this study is to evaluate and enhance the performance of YOLOv8s in detecting 3 types of sporangia associated with *Plasmopara viticola*, the pathogen of grapevine downy mildew: normal, malformed, and empty sporangia. We curated a comprehensive dataset of grapevine downy mildew sporangia images to facilitate this evaluation. In addition, we improved upon the YOLOv8s model by refining its backbone, neck, and loss function, resulting in an enhanced version named Adaptive Cross Fusion-YOLOv8s (AFM-YOLOv8s). The goal of AFM-YOLOv8s is to achieve a balanced accuracy and speed in detecting these types of sporangia, thereby serving as a valuable tool for analyzing images captured by automatic spore traps and facilitating large-scale monitoring of disease infection risks.

## Materials and Methods

### Experimental design

**Fig. 1.**
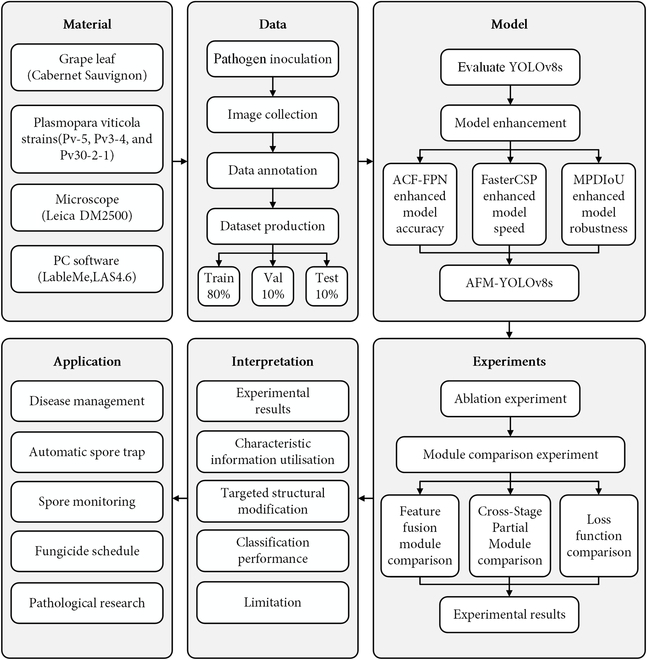
Experimental, technical design, and workflows.

This study is structured into 6 major sections, as illustrated in Fig. 1. Initially, we assembled the requisite materials, including grape leaf substrate, strains, microscope, and software, for tasks such as pathogen inoculation, fungicide treatment, observation, image capture, and labeling. Subsequently, we devised, developed, and trained our object detection model to identify sporangia in diverse forms. We conducted experiments to assess and compare the performance of our model with other state-of-the-art models. The comprehensive results of these experiments are outlined in Results. Finally, we analyzed the findings, pointed out the limitations, and explored potential applications of our developed models. The data and code utilized in this study can be accessed via https://github.com/Lzzyyy123/spore_detect.

### Grapevine downy mildew and its sporangia variants

The sporangia of *P. viticola* play a crucial role in the rapid spread and infection process of grapevine downy mildew [19]. In fungicide resistance studies, these sporangia are often treated with different fungicides, resulting in sporangia’ variations in forms including normal, malformed, and empty. Normal sporangia are capable of germination and causing infection under suitable environmental conditions. They have a typical appearance and size specific to *P. viticola*, and their successful germination is crucial for the spread of downy mildew in vineyards. Malformed sporangia may have irregular shapes, sizes, or structures and are often less viable or nonviable, meaning that they may not germinate properly or at all, reducing the spread of disease. Empty sporangia are the ones that appear to be devoid of content, essentially hollow, which is thought to be zoospores that have been released [2]. Empty sporangia are nonviable and cannot contribute to the disease cycle. The presence of empty sporangia can be indicative of stress factors affecting the pathogen, such as adverse environmental conditions, disease management practices, or inherent weaknesses in the pathogen population. Understanding the different forms of grape downy mildew sporangia and their viability can be essential for managing this disease and studying fungicide efficacy against different strains.

### Data acquisition

To obtain images of grapevine downy mildew sporangia of the mentioned forms, a range of materials and tools were utilized, including a grape variety (Cabernet Sauvignon), test *P. viticola* strains (Pv-5, Pv3-4, and Pv30-2-1), a microscope image collection instrument, and image processing software. The Cabernet Sauvignon grape was sourced from the experimental base of the Guangxi Academy of Agricultural Sciences. The *P. viticola* strains (Pv-5, Pv3-4, and Pv30-2-1) were isolated and stored by the Guangxi Crop Genetic Improvement and Biotechnology laboratory. The microscope image collection instrument used was the Leica DM2500 fluorescent microscope equipped with a Leica MC170 high-resolution digital camera capable of capturing real-time images with a resolution of 5 million pixels. Image acquisition was facilitated using LAS4.6 software.

The microscopic image acquisition procedure involved following steps (Fig. S1). First, young leaves of Cabernet Sauvignon were cleaned twice with sterile water and blot-dried with filter paper. Subsequently, the leaves were positioned in a petri dish with fully moistened filter paper on the reverse side, and the abaxial surfaces of young leaves were inoculated with 30-μl droplets of *P. viticola* sporangia suspension (10^5^ sporangia/ml). The droplets on the leaves were then removed overnight using soft paper, and the leaves were placed in the incubator until new downy mildew grows.

Following the preparation of suspensions, obtained by removing filaments using single-layer lens paper and quantified using a cell counting board, the suspension was diluted with sterile water to achieve concentrations of 1 × 10^5^, 1 × 10^6^, and 1 × 10^8^ sporangia/ml. Subsequently, to capture microscopic images of sporangia under fungicide application, the sporangium suspensions were mixed with 72.2% propamocarb hydrochloride (diluted 6,000 times), 32.5% difenoconazole azoxystrobin (diluted 6,000 times), and 80% ethylicin (diluted 6,000 times) in a 1:1 ratio, respectively, while sterile water served as the blank control. Thirty microliters of the mixture were then dropped onto the center of concave slides and covered. The slides were incubated in a 4 °C chamber under dark conditions for 1 h, followed by culturing at 22 °C for 3 to 4 h. A total of 100 slides were prepared for analysis. To observe sporangium germination, the slides were placed on a DM2500 microscope. Target field images were captured at 10 × 20 and 10 × 40 magnification after locating specific areas using a low-power objective. Twenty random field images were captured for each slide using LAS4.6, with a resolution of 2,592 × 1,944, and saved in “.tif” format for further analysis and documentation.

### Labeling imageries of sporangia

**Fig. 2.**
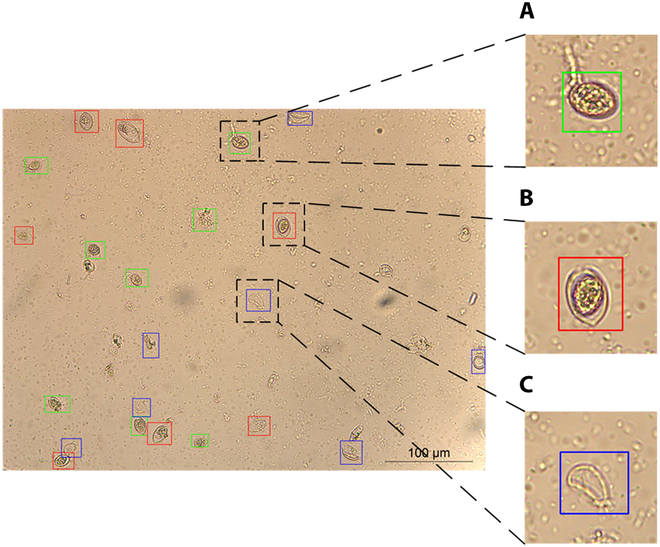
Example of 3 forms of sporangia under the treatment of Propamocarb. (A) Normal, characterized by intact cell walls and full internal material. (B) Malformed, a sporangium that deviates from the expected or typical characteristics, possibly due to genetic variation, environmental stress, or other factors resulting in irregularities in morphology, size, color, or other features. (C) Empty, characterized by complete release of internal material and a translucent state.

We utilized the LabelMe tool (available at https://github.com/wkentaro/labelme) for sporangia bounding box delineation. Each sporangium within each image was manually encircled with a bounding box. This process involved visually inspecting each sporangia object and drawing a rectangular box that closely fitted the contours, ensuring minimal background inclusion within the box to reduce potential classification errors during model training. Following the bounding box annotation, each sporangium was assigned 1 of 3 classes (normal, malformed, and empty) according to its morphological characteristics, determined by expert’s judgments. Figure 2 illustrates an example of the image containing labels 3 types of sporangia of downy mildew.

**Fig. 3.**
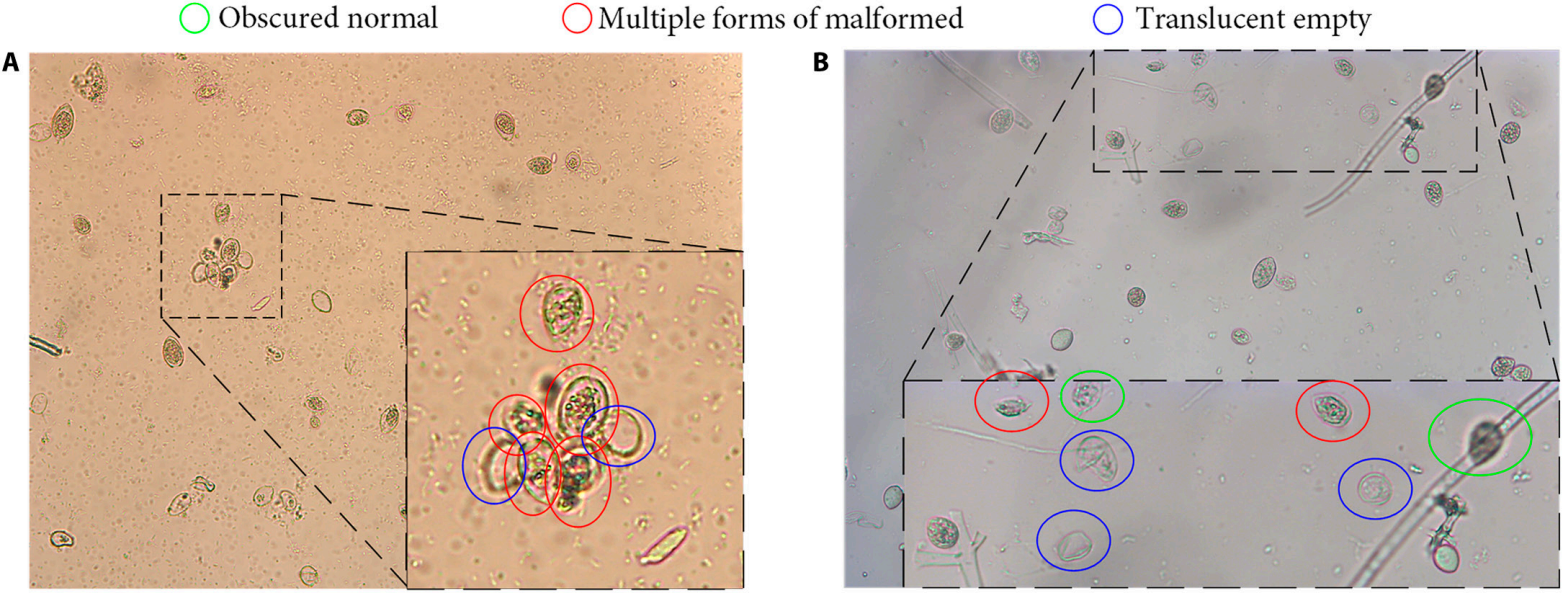
The characteristics of the 3 types of sporangia. (A) Sporangia with small-size, complex background that were generated by application of propamocarb fungicide. (B) Sporangia with obscured, incomplete, and polymorphic forms. Red circles indicate multiple forms of malformed sporangium labels, green ones indicate obscured normal sporangia labels, and blue ones indicate translucent empty sporangia.

Notably, the dataset, as depicted in Fig. 3, shows several key complexities for detection: impurities and distracting elements alongside densely clustered sporangia (Fig. 3A), obscured or polymorphic sporangia (Fig. 3B), and substantial morphological differences even within the same label. These complexities, including sporangia aggregation and distribution at image edges, pose challenges for feature fusion networks and introduce occlusion-related complexities.

To ensure a high-quality dataset, we removed images that either lacked targets or were excessively blurred. The final dataset comprises a total of 1,619 sporangium images, which were randomly divided into 3 distinct groups: 80% for training, 10% for validation, and 10% for testing, as detailed in Table 1, where the number of sporangium instances per category was obtained using a Python program we developed, which is available in our GitHub repository. To mitigate the problem of overfitting, online data augmentation techniques including mosaic and HSV (hue, saturation, and value) conversion, along with horizontal and vertical flipping, were carried out dynamically to enhance the dataset’s diversity and robustness [20].

**Table 1.** Distribution of sporangium images across training, validation, and testing sets and the counts of normal, malformed, and empty sporangia in each set.

Name	Images	Total	Normal	Malformed	Empty
Train	1,299	27,677	13,953	5,123	8,600
Validation	160	2,490	1,414	365	711
Test	160	3,020	1,887	355	778

### AFM-YOLOv8s

**Fig. 4.**
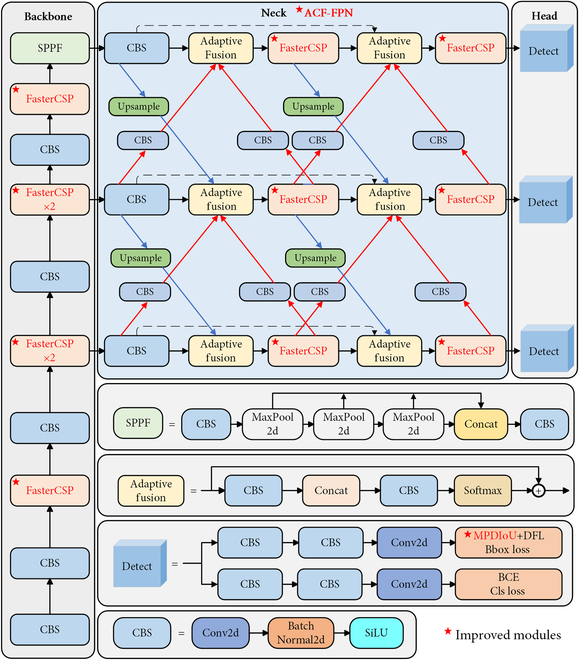
The architecture of AFM-YOLOv8s network. Red stars represent improved modules, including FasterCSP, ACF-FPN, and MPDIoU.

YOLOv8s, a lightweight version of YOLOv8 that has an architectural framework of 3 main components: the backbone network (Fig. 4, left), neck network (Fig. 4, middle), and detection head (Fig. 4, right) [15]. The backbone network comprises Convolution-BatchNorm-SiLU (CBS) and Spatial Pyramid Pooling-Fast (SPPF) modules, responsible for extracting feature representations. The neck network is responsible for integrating, refining, and consolidating the feature information extracted by the backbone network and passing the results to the detection head. The detection head uses binary cross-entropy (BCE) classification loss, distribution focal loss, and bounding box regression loss with Complete Intersection over Union (CIoU), generating the final detection results by leveraging feature information. During our evaluation of using YOLOv8s for detecting sporangia with diverse morphological variations and instances of occlusion, we identified limitations in balancing speed and accuracy. To overcome these challenges, we developed the AFM-YOLOv8s model (Fig. 4). AFM-YOLOv8s preserves the fundamental architectural framework of YOLOv8s, while incorporating 3 enhancements [Faster Cross-Stage Partial Module (FasterCSP), Adaptive Cross Fusion Feature Pyramid Network (ACF-FPN), and Minimum Point Distance IoU (MPDIoU)] as denoted by red stars in Fig. 4.

#### FasterCSP for a lighter feature representation extraction model

**Fig. 5.**
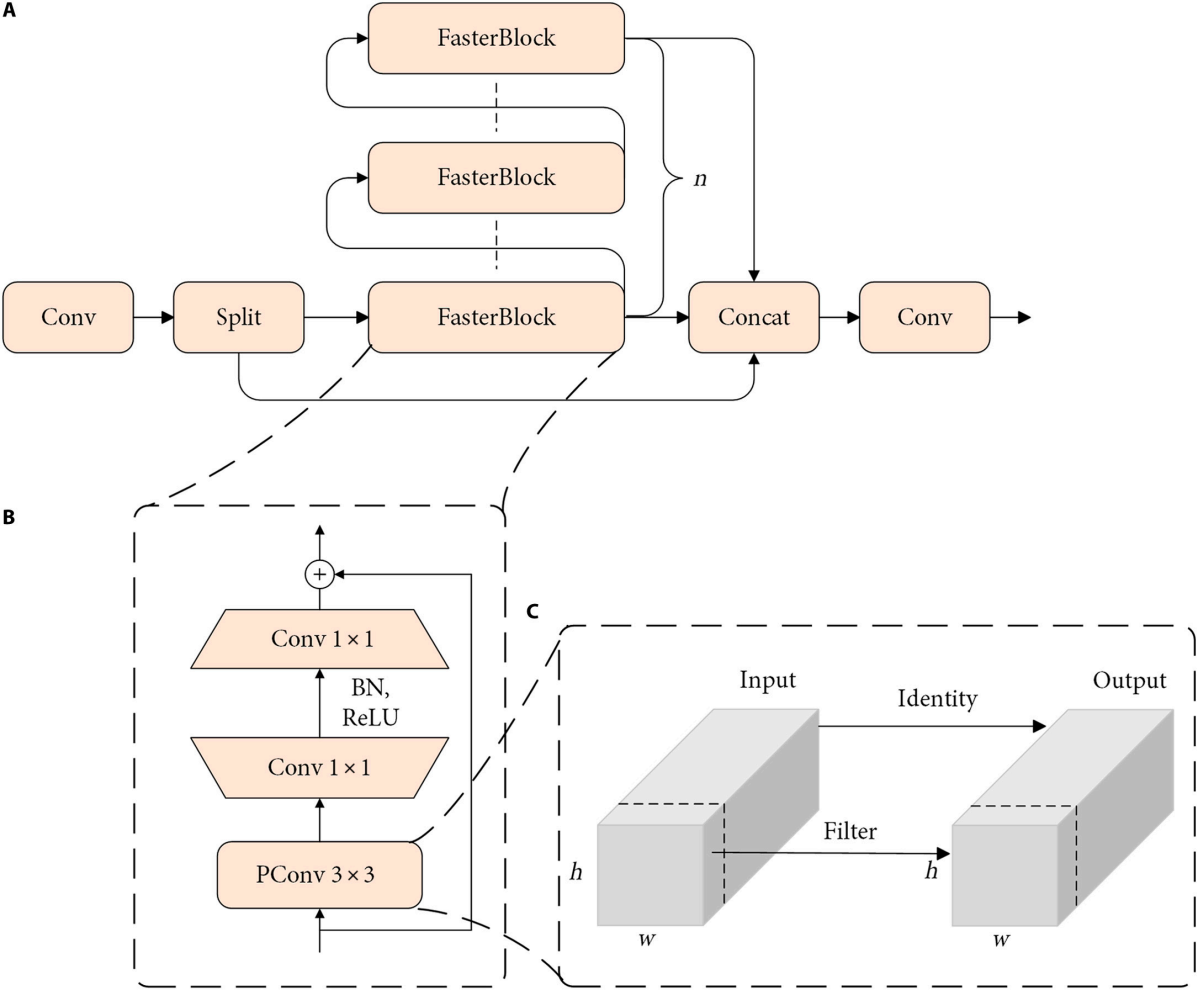
The structure of FasterCSP (A), FasterBlock (B), and PConv (C). FasterBlock is a submodule of FasterCSP, and PConv is a submodule of FasterBlock.

Within YOLOv8, the C2f module is realized by connecting multiple bottleneck modules serially, with each bottleneck module comprising 2 convolutional blocks. It enables the C2f module to learn rich feature representations but increases the computational burden and complexity of the model. Following [21], a lightweight module FasterBlock was introduced to replace the bottleneck module to reduce computational demands. This integration resulted in FasterCSPs, aimed at optimizing models’ computational efficiency and performance (Fig. 5A). The FasterBlock is composed of a Partial Convolution (PConv) layer coupled with two 1 × 1 convolution layers, Batch Normalization (BN), and rectified linear unit (ReLU) activation (Fig. 5B). PConv substantially contributes to the lightweight of FasterBlock by conducting regular convolution operations on only a subset of input channels to extract spatial features while preserving the integrity of the remaining channels. While PConv module ensures that the input and output feature maps maintain an identical number of channels, it does not sacrifice essential information (Fig. 5C).

#### ACF-FPN for enhanced feature representation integration

**Fig. 6.**
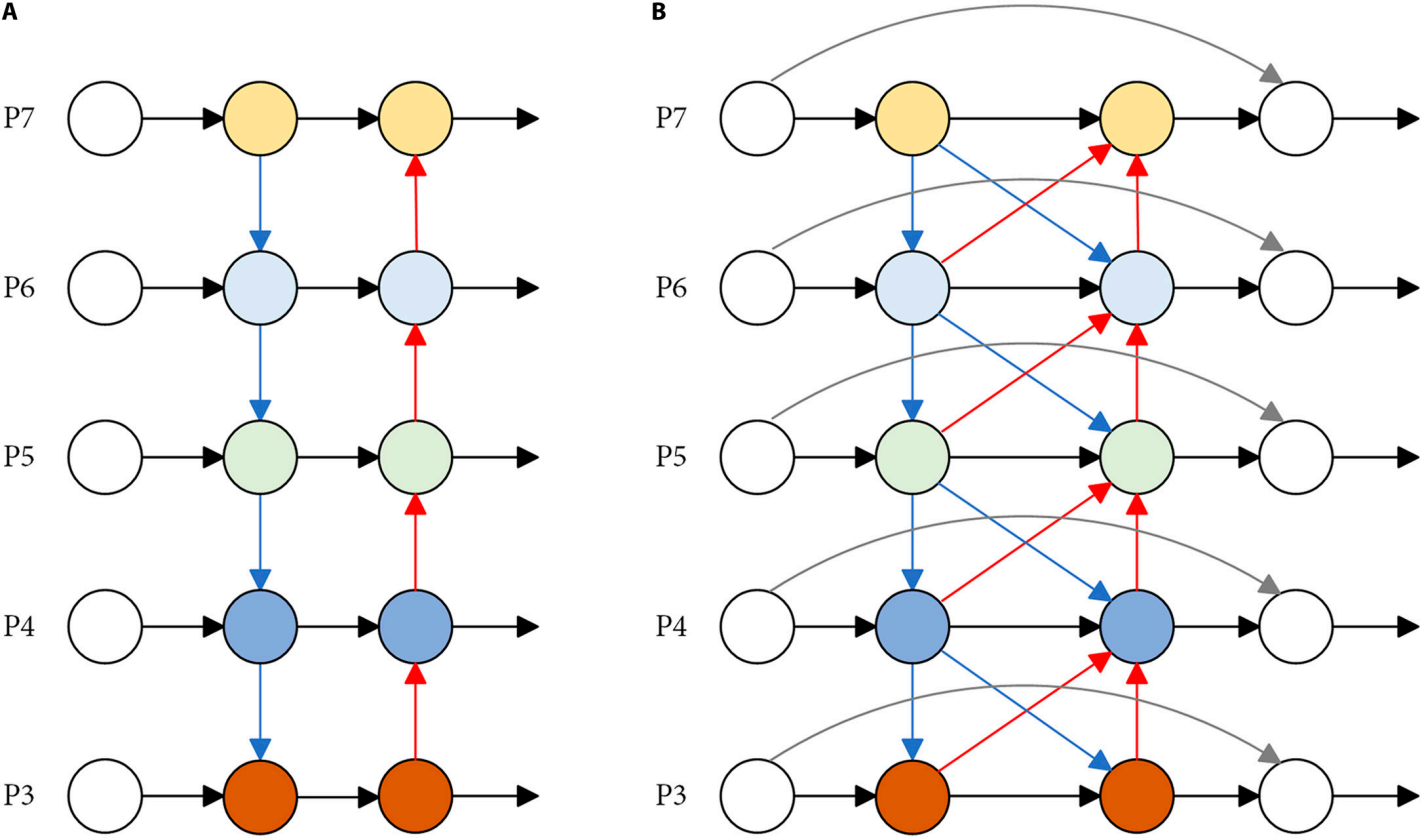
The structure of ACF-FPN. P3 to P7 represent the multiscale features of layers 3 to 7 from low to high levels. (A) Top-down path augmentation for feature fusion in PANet constructed and bottom-up path for feature fusion. (B) ACF-FPN with bidirectional connectivity, adaptive fusion, and residual connectivity added compared to PANet.

YOLOv8’s Path Aggregation Network (PANet) neck refers to FPN. It introduces a bottom-up PANet based on the traditional top-down feature fusion path (Fig. 6A) [22]. The design aims to enhance the feature information in lower-level dimensions. PANet requires adjustment of feature maps to a uniform dimension for summation during information propagation and interaction, which potentially leads to information conflicts among features of different dimensions and thereby limiting the comprehensive utilization of multiscale features [23]. To address this issue, we created an innovative feature fusion pyramid called ACF-FPN. This network aims to enhance the extraction of semantic information from high-level features through bidirectional cross connectivity while preserving the details of low-level features (Fig. 6B).

**Fig. 7.**
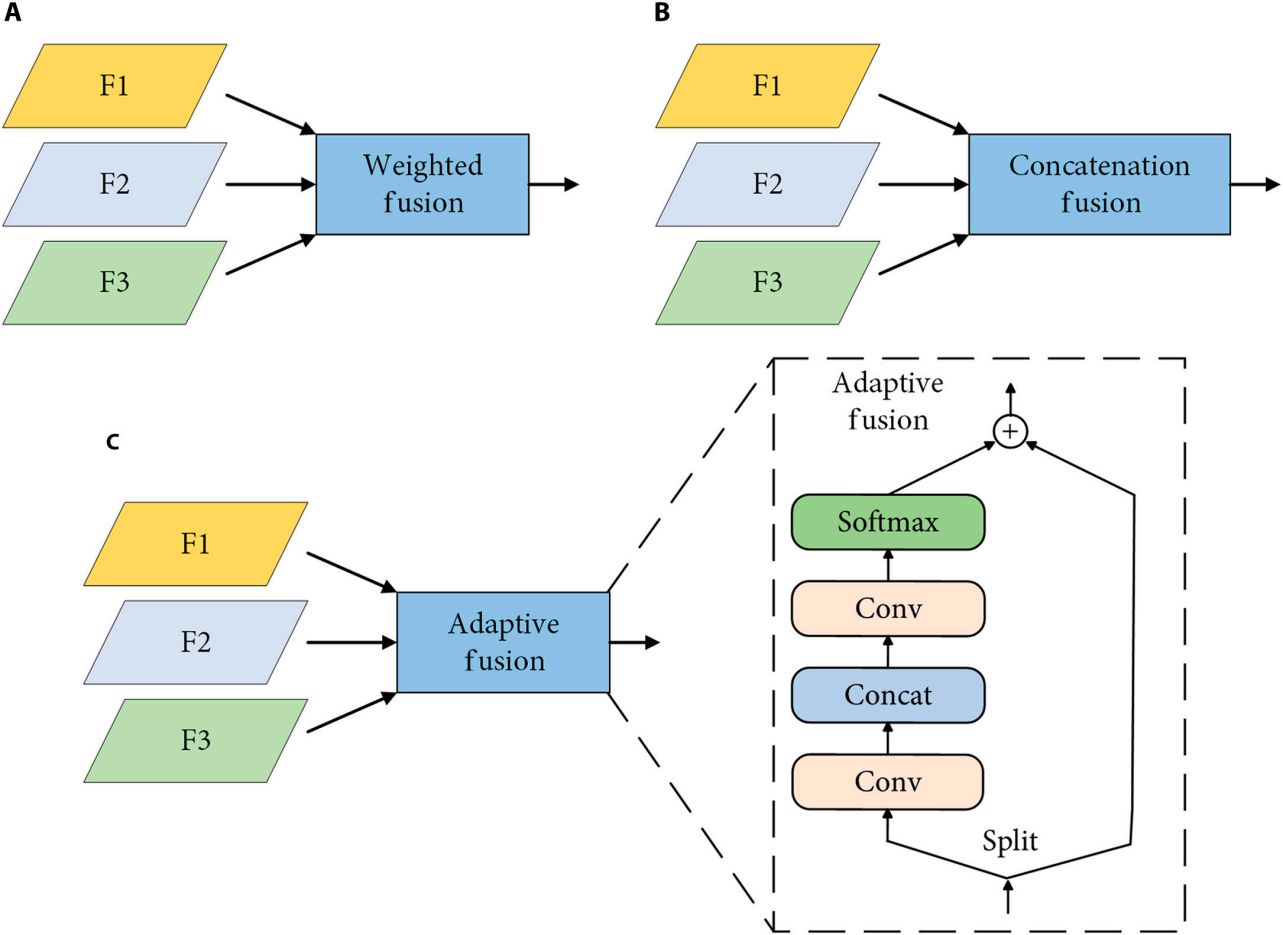
Three fusion methods, where F1, F2, and F3 represent different feature inputs. (A) Weighted fusion. (B) Fusion with sequence concatenation. (C) Fusion with spatial adaptive weights.

The design of the ACF-FPN framework encompasses several noteworthy aspects. First, to tackle the semantic discrepancy between nonadjacent levels that can lead to information conflict during direct fusion, ACF-FPN increases the bidirectional fusion path between neighboring layers as shown by the red and blue arrows in (Fig. 6B), where the blue arrows represent the upsampling and the red arrows represent the downsampling using convolutional implementation. Second, to avoid information loss and to preserve details from being overwhelmed by conflicting information generated during the FPN message transfer process, we created residual links between the input nodes and the corresponding output nodes in the same layer, as shown by the curved arrows in (Fig. 6B). Moreover, concerning the fusion methods for integrating features of varying resolutions at each node, 3 common techniques are typically used, as depicted in Fig. 7. While the weighted fusion method and the concatenation fusion method directly incorporate features in spatial and channel dimensions, potentially leading to conflicts in linguistic information, the adaptive fusion method adopts spatially adaptive weights through convolutional connections and Softmax operations. This approach allocates higher weights to feature categories prone to information loss during fusion, thus fostering a balanced consideration of different feature categories and reducing potential information conflicts [24]. Consequently, adaptive fusion is utilized for cross-scale fusion in ACF-FPN. In addition, as shown in the ACF-FPN structure in Fig. 4, we utilized 3 CBS modules to unify the channel numbers of feature maps at different scales extracted by the backbone network, thereby reducing the complexity of the entire ACF-FPN module.

#### MPDIoU-based loss function

**Fig. 8.**
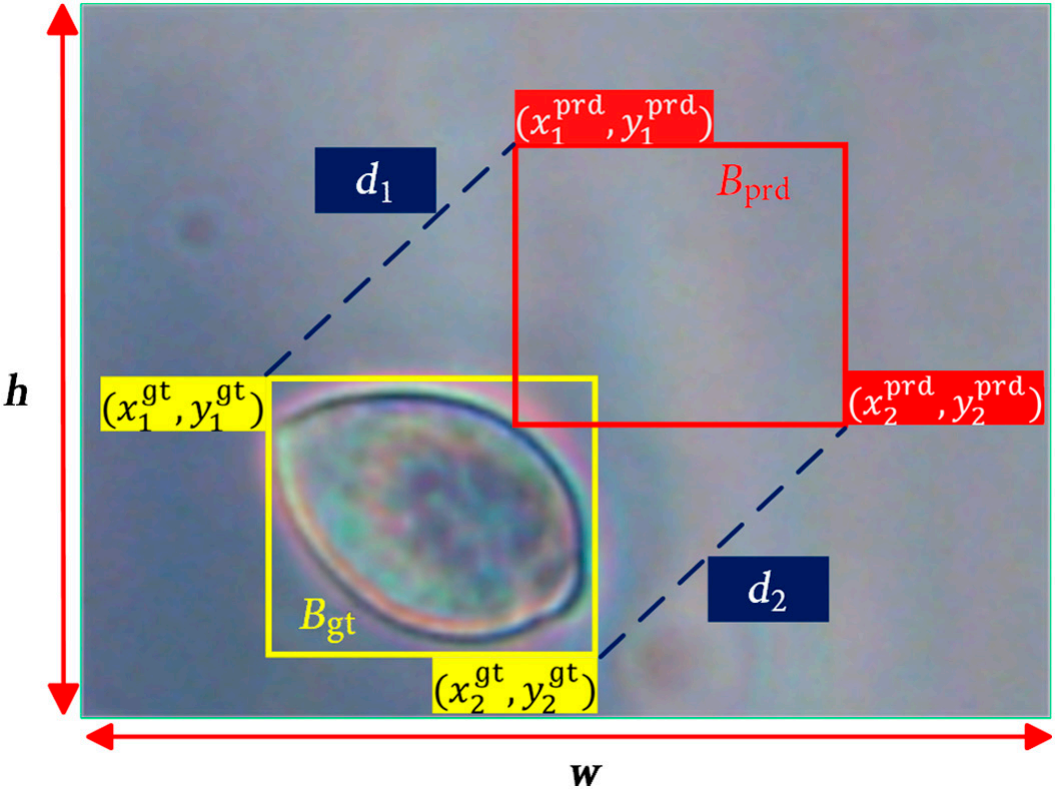
Geometric illustration for computing $L_{\text{MPDIoU}}$. The yellow and red boxes represent the ground-truth and the predicted bounding box, respectively. The *d*_1_ and *d*_2_ represent the distance between the corners of the predicted and the ground-truth bounding box, respectively. The *h* and *w* represent the height and width of the input feature map, respectively.

In YOLOv8, the loss function $L_{\text{CIoU}}$ quantifies the accuracy of bounding box regression by considering the distance between the centroids of predicted and ground-truth boxes, along with the aspect ratio [25]. Since the aspect ratio of $L_{\text{CIoU}}$ is a relative value, its efficiency gets lower when the predicted frame shares the same aspect ratio as the real frame but has markedly different widths and heights [26]. The reason is that geometric variations in the dimensions of sporangia lead to a sharp decrease in the accuracy of bounding box regression. To address this issue, $L_{\text{CIoU}}$ is replaced by $L_{\text{MPDIoU}}$, which utilizes the minimum point distance between the predicted bounding box and the ground-truth bounding box rectangle to compute boundary similarity (Fig. 8) [26]. $L_{\text{MDPIoU}}\;$is calculated as:

$$L_{\text{MPDIoU}}=1-\text{MPDIoU}$$ (1)

where MPDIoU is calculated as:

$$\text{MPDIoU}=\text{IoU}-\frac{d_{1}^{2}}{h^{2}+w^{2}}-\frac{d_{2}^{2}}{h^{2}+w^{2}}$$ (2)

where IoU metric quantifies the ratio between the area of intersection and the area of union of the predicted bounding box and the ground-truth bounding box, *d*_1_ and *d*_2_ denote the distances between the coordinates of the upper-left and lower-right corners of the minimization prediction bounding box $B_{\text{prd}}$ and the ground-truth bounding box $B_{\mathrm{gt}}$, as shown by the blue dashed lines in Fig. 8. In addition, the symbols *h* and *w* signify the width and height of the input feature map images, as indicated by the red arrows in Fig. 8.

In the calculation of $L_{\text{MPDIoU}}$, IoU should be calculated first as:

$$\mathrm{IoU}=\frac{B_{\mathrm{gt}}\cap B_{\mathrm{prd}}}{B_{\mathrm{gt}}\cup B_{\mathrm{prd}}}$$ (3)

where $B_{\mathrm{gt}}$ and $B_{\mathrm{prd}}$ denote the ground-truth bounding box and prediction bounding box, respectively, as illustrated in Fig. 8 within the yellow and red boxes.

### Experimental design

#### Ablation experiments

The proposed model consists of 3 components, namely, FasterCSP, ACF-FPN, and the $\;L_{\text{MPDIoU}}$ loss function. An ablation study contrasting these components against the foundational YOLOv8 model was conducted to evaluate their individual contributions. First, we replaced the PANet neck module with ACF-FPN. Then, we added the replacement for C2f module in the model’s backbone and neck network with FasterCSP. Finally, the loss function $L_{\text{MPDIoU}}$ was also included.

#### Module comparison

To evaluate ACF-FPN’s capability in feature fusion, we compared it with YOLOv8s baseline model, YOLOv8s-AFPN [27], YOLOv8s-GFPN [28] and YOLOv8s-BiFPN [29]. Metrics of mAP50 (mean average precision at 50% IoU; Eq. 7), F1 score (harmonic mean of precision and recall; Eq. 8), FPS (frames per second), parameter size (total number of trainable parameters in the model), GFLOPs (giga floating point operations), and model size (disk space required to store the mode) were used in the evaluation.

Further, to assess the effectiveness of the FasterCSP module, we replaced the YOLOv8-ACFFPN model’s C2f components within both the backbone and neck with FasterCSP, comparing it with YOLOv8s-ACFFPN, YOLOv8s-ACFFPN-GhostCSP [30], YOLOv8s-ACFFPN-VoVGSCSP [31]. Metrics of mAP50, F1 score, FPS, GFLOPs, parameter size, and model size were used in the evaluation. This set of experiments was designed to pinpoint the most efficient cross-stage partial network configuration.

Last, the influence of the $L_{\text{MPDIoU}}$ loss function on bounding box detection accuracy was scrutinized. The AFM-YOLOv8s model underwent testing with various loss functions including $L_{\text{CIoU}}$, $L_{\text{DIoU}}$, $L_{\text{GIoU}}$, and $L_{\text{MPDIoU}}$ to assess their impact on model performance during both training and validation phases, thereby determining the optimal approach for bounding box regression.

#### Multiscale experiments

To evaluate the performance of the AFM-YOLOv8s algorithm for multiscale sporangia detection, 2 experiments were designed. The first is a comparison experiment on sporangia detection based on current data acquired by microscope, under 2 magnifications of 10 × 20 and 10 × 40. The test dataset was divided into subsets according to the magnifications. We assessed the algorithm’s performance using mAP50, precision, recall, and F1 score. However, because of the unequal sample sizes and different sporangia collection regions in the 10 × 20 and 10 × 40 subsets, such an experiment could not accurately reflect the algorithm’s multiscale detection performance.

Therefore, we designed the second experiment. For this experiment, the images were acquired by splitting 92 images, originally at a 100-μm scale and containing 2,481 sporangia, into 4 equal subimages each. These subimages were then enlarged to the original size before splitting, resulting in a total of 368 images at a 50-μm scale, containing a total of 2,765 sporangia, representing the same regions (see Fig. S2 for the workflow of multiscale comparison image generation). Because sporangia at the splitting edges were split into multiple parts, belonging to multiple images, the total number of sporangia at the 50-μm scale is higher than at the 100-μm scale. We evaluated the multiscale detection performance of AFM-YOLOv8s using mAP50, precision, recall, and F1 score.

#### Model comparison

To assess the efficacy of AFM-YOLOv8s algorithm for sporangia detection, we conducted a comparative analysis against 6 leading detection models, spanning both 1-stage and 2-stage approaches. The benchmarked models in this comparison were Faster R-CNN [32], RetinaNet [33], RT-DETR [34], YOLOv3-tiny [35], YOLOv5s, and YOLOv8s, chosen for their proved high performance in various detection tasks. The models utilized the following backbone networks for feature extraction: ResNet50, a classic residual network known for its excellent feature representation; Darknet53, designed for computational efficiency and high performance; and CSPDarknet53, which incorporates cross-stage partial connections to improve feature reuse and computational efficiency. These backbone networks are widely recognized for their outstanding performance. Metrics of mAP50, F1 score, FPS, parameter size, detection time (time to detect one image), and model size were used in the evaluation. This evaluation aims to validate the advantages and improvements that AFM-YOLOv8s brings to the domain of sporangia detection.

### Test environment

Our experimental setup used an NVIDIA GeForce RTX A5000 graphic processing unit and an Intel Xeon Gold 6330 central processing unit, running on an Ubuntu operating system with PyTorch as the deep learning framework. To maintain consistency across experimental results, the network’s model parameters were standardized. The optimization strategy used was stochastic gradient descent, with an initial learning rate of 0.01. In addition, we implemented a weight decay coefficient of 0.0005 to prevent overfitting and set the batch size for training at 16. The model underwent a warm-up phase for 3 epochs to gradually adjust to the optimal learning rate, followed by a comprehensive training phase spanning 300 epochs. All training images were resized to a uniform input size of 640 pixels × 640 pixels to standardize the input data format for the network.

### Evaluate metrics

In this study, metrics such as precision, recall, mAP50, and F1 score were used to evaluate the accuracy of each model. Precision and recall denote the likelihood of accurately identifying a positive sample and the probability of detecting a positive sample among actual positive samples, respectively. Precision and recall can be calculated in Eqs. 4 and 5, respectively.

$$\text{Percision}=\frac{\mathrm{TP}}{\mathrm{TP}+\mathrm{FP}}$$ (4)

$$\text{Recall}=\frac{\mathrm{TP}}{\mathrm{TP}+\mathrm{FN}}$$ (5)

where TP, FP, and FN represent true positive, false positive, and false negative respectively. However, relying solely on precision and recall cannot comprehensively assess the accuracy of detection. Therefore, mAP50 and F1 score were introduced to more fully assess the effectiveness of the detection algorithm. The mAP50 refers to the average precision at an IoU threshold of 0.5 (AP50) value calculated on the basis of the IoU between the predicted bounding box and the ground-truth bounding box. The AP50 metric is calculated by integrating the area under the precision–recall curve from 0 to 1, with the IoU threshold set at 0.5. AP50 and mAP50 can be calculated as:

$$\mathrm{AP}50=\int_{0}^{1}\;\text{Percision}\left(\text{Recall}\right)d\text{Recall},\mathrm{loU}\geq 0.5$$ (6)

$$\mathrm{mAP}50=\frac{\sum_{i=1}^{n}\;\mathrm{AP}50_{i}}{n}$$ (7)

where *n* is the number of classes. The F1 score can be calculated as:

$$F1=\left(\frac{2\times \text{Percision × Recall}}{\text{Percision}\;+\;\text{Recall}}\right)\times 100\%$$ (8)

In addition, metrics such as the size of the model, number of parameters, image detection time, and FPS are also used to evaluate the speed of the model.

## Results

### Ablation experiments

The YOLOv8s obtained an mAP50 of 88.6% and an F1 score of 83.2% (Table 2). With the integration of ACF-FPN into the model, a notable improvement, with mAP50 increasing by 2.3% and the F1 score rising by 3.0%, was achieved. In addition, the model’s overall parameters and GFLOPs decreased by 2.5% and 0.8, respectively. However, a reduction of FPS from 106.5 to 74.7 was observed. In corporation of FasterCSP, replacing the C2f modules in both the backbone and neck showed a minor decline in performance, with mAP50 and F1 score decreased by 0.3% and 1.1%, respectively. This change reduced the model’s complexity, as evidenced by decreases in the number of parameters (reduced by 1.76 × 10^6^), GFLOPs (reduced by 6.1), and model size (reduced by 3.4 MB). When  $\;L_{\text{MPDIoU}}$ was also introduced, the number of parameters, the GFLOPs, and the model size of the model achieved no obvious changes, while mAP50, F1 score, and FPS were improved by 0.7%, 0.2%, and 0.6 (frames/s), respectively. Across these modifications, the FPS experienced an initial notable drop, followed by a marginal recovery, underscoring the balance between computational efficiency and detection performance. Despite fluctuations in FPS, the enhancements contributed to overall gains in detection accuracy, as evidenced by improvements in mAP50 and F1 scores.

**Table 2.** Results of ablation experiments with metrics of mAP50 (Eq. 7), F1 score (Eq. 8), FPS, parameter size, GFLOPs, and model size. Note that bold font indicated the optimal result.

ACF-FPN	FasterCSP	$L_{\text{MPDIoU}}$	mAP50	F1	FPS (frames/s)	Parameter size (×10^6^)	GFLOPs	Model size (MB)
			0.886	0.832	**106.5**	11.13	28.4	21.5
√			0.909	**0.862**	74.7	8.63	27.6	16.8
√	√		0.906	0.851	75.0	**6.87**	**21.5**	**13.4**
√	√	√	**0.913**	0.853	75.6	**6.87**	**21.5**	**13.4**

### Comparison of different feature fusion modules

Among all the fusion modules, ACF-FPN achieved the highest mAP50 and F1 scores, with values of 90.9% and 86.2%, respectively (Table 3). Conversely, the baseline YOLOv8s model excelled in computational efficiency, leading with the highest FPS at 106.5. Among the fusion modules evaluated, bidirectional weighted FPN (BiFPN) distinguished itself by minimizing the model’s complexity, evidenced by having the lowest counts in model parameters (7.37 × 10^6^), GFLOPs (25.0), and model size (14.3 MB), suggesting an optimized balance between performance and computational resource requirements.

**Table 3.** Comparison of 5 different feature fusion modules with metrics of mAP50 (Eq. 7), F1 score (Eq. 8), FPS, parameter size, GFLOPs, and model size. Note that bold font indicated the optimal result.

Models	mAP50	F1	FPS (frames/s)	Parameter size (×10^6^)	GFLOPs	Model size (MB)
YOLOv8s	0.886	0.832	**106.5**	11.13	28.4	21.5
YOLOv8s-AFPN	0.903	0.850	70.7	8.86	25.1	17.3
YOLOv8s-GFPN	0.899	0.845	78.7	12.25	29.7	24.8
YOLOv8s-BiFPN	0.901	0.853	104.9	**7.37**	**25.0**	**14.3**
YOLOv8s-ACFFPN	**0.909**	**0.862**	74.7	8.63	27.6	16.8

### Comparison of different cross-stage partial network modules

All cross-stage partial network modules could markedly reduce the number of parameters and GFLOPs (Table 4). Among them, GhostCSP was the most efficient, achieving the lowest values in both parameters and GFLOPs. However, this efficiency came at the cost of reduced accuracy, as indicated by lower mAP50 and F1 scores. In comparison to the baseline model, VoVGSCSP showed the most substantial drop in performance, with mAP50 and F1 scores declining by 0.9% and 1.3%, respectively. On the other hand, FasterCSP presented a minimal reduction in mAP50 by only 0.3%, demonstrating its ability to maintain closer accuracy levels to the baseline while improving operational speed, as evidenced by the highest FPS rate of 75.

**Table 4.** Comparisons of the 4 cross-stage partial network modules with metrics of mAP50 (Eq. 7), F1 score (Eq. 8), FPS, parameter size, GFLOPs, and model size. Note that bold font indicated the optimal result.

Models	mAP50	F1	FPS (frames/s)	Parameter size (×10^6^)	GFLOPs	Model size (MB)
YOLOv8s-ACFFPN	**0.909**	**0.862**	74.7	8.63	27.6	16.8
YOLOv8s-ACFFPN-GhostCSP	0.903	0.848	59.8	**6.08**	**18.9**	**12.0**
YOLOv8s-ACFFPN-VoVGSCSP	0.9	0.849	53.3	7.82	21.4	15.4
YOLOv8s-ACFFPN-FasterCSP	0.906	0.851	**75.0**	6.87	21.5	13.4

### Improvements made by MPDIoU loss function

Table 5 presents the experimental results with different loss functions. Among them, MPDIoU achieved the highest scores for mAP50, precision, and F1, with values of 0.913, 0.871, and 0.853, respectively. DIoU, on the other hand, attained the highest recall score of 0.866.

**Table 5.** Comparison of 4 different loss functions with metrics of mAP50 (Eq. 7), precision (Eq. 4), recall (Eq. 5), and F1 score (Eq. 8). Note that bold font indicated the optimal result.

Models	mAP50	Precision	Recall	F1
YOLOv8s-ACFFPN- FasterCSP-CIoU	0.906	0.86	0.842	0.851
YOLOv8s-ACFFPN- FasterCSP-DIoU	0.906	0.828	**0.866**	0.847
YOLOv8s-ACFFPN- FasterCSP-GIoU	0.893	0.822	0.863	0.842
YOLOv8s-ACFFPN- FasterCSP- MPDIoU	**0.913**	**0.871**	0.835	**0.853**

The experimental results using different loss functions in model training and validation are shown in Fig. S3. Throughout the training of the enhanced YOLOv8s network, all 4 loss functions markedly diminished the model’s boundary regression loss. In the model validation phase, the loss values for the initial 100 epochs exhibited noticeable fluctuations across all 4 loss functions before stabilizing. Notably, the GIoU loss function produced comparatively elevated training and validation loss values, whereas MPDIoU loss function achieved the lowest such values.

### Performance of AFM-YOLOv8s for multiscale detection

Table 6 presents the detection results for AFM-YOLOv8s at different magnifications. The model performs significantly better at 10 × 20 magnification than at 10 × 40, with an average mAP50 for 10 × 20 images being 8% higher than that for 10 × 40 images. In addition, 10 × 20 images show superior precision, recall, and F1 scores. This performance difference is primarily due to the higher number of sporangia instances in 10 × 20 images and the different size of the covering region, whereas the increased magnification rate (10 × 40) results in fewer sporangia instances per image.

**Table 6.** Performance of AFM-YOLOv8s at different magnification subsets of the test set with metrics of mAP50 (Eq. 7), precision (Eq. 4), recall (Eq. 5), and F1 score (Eq. 8).

Magnification rate	Number of images	Categories	Instances	mAP50	Precision	Recall	F1
10 × 20	92	Normal	1492	0.984	0.945	0.962	0.953
		Malformed	327	0.852	0.830	0.719	0.771
		Empty	662	0.933	0.874	0.847	0.860
		All	2481	0.923	0.883	0.842	0.862
10 × 40	68	Normal	395	0.963	0.904	0.933	0.918
		Malformed	28	0.649	0.685	0.607	0.644
		Empty	116	0.918	0.896	0.759	0.822
		All	539	0.843	0.829	0.766	0.796

Table 7 presents the results of the multiscale experiments. The experimental results show that the model performs slightly better at the 50-μm scale compared to the 100-μm scale. Specifically, when the average metrics of the 3 sporangia types are considered (with the categories labeled as All in Table 7), the mAP50, precision, recall, and F1 scores of the model with a 50-μm scale are 0.8%, 1.8%, 3.6%, and 2.7% higher, respectively, than those of the model with a 100-μm scale. As the image scale increases to 100 μm, larger image pixels allow the model to capture finer details and features. Conversely, at a 50-μm scale, the model focuses on more instances and a broader range of features. Despite a slight increase in accuracy when downscaling, AFM-YOLOv8s model achieve high accuracy consistently across different scales, effectively detecting sporangia.

**Table 7.** Performance of AFM-YOLOv8s vary at scale changes with metrics of mAP50 (Eq. 7), precision (Eq. 4), recall (Eq. 5), and F1 score (Eq. 8)

Scale	Number of images	Categories	Instances	mAP50	Precision	Recall	F1
100 μm	92	Normal	1492	0.984	0.945	0.962	0.953
		Malformed	327	0.852	0.830	0.719	0.771
		Empty	662	0.933	0.874	0.847	0.860
		All	2481	0.923	0.883	0.842	0.862
50 μm	368	Normal	1714	0.931	0.947	0.915	0.931
		Malformed	332	0.885	0.833	0.826	0.829
		Empty	719	0.949	0.923	0.893	0.908
		All	2765	0.931	0.901	0.878	0.889

### Comparison with other state-of-the-art models

Regarding mAP50 value and F1 scores, AFM-YOLOv8s achieved the highest values of 91.3% and 85.3%, respectively (Table 8). Faster R-CNN, YOLOv3-tiny, YOLOv5s, and YOLOv8s had similar detection accuracies, with mAP50 of 87.4%, 87.6%, 88.5%, and 88.6%, respectively. In terms of the number of parameters and model occupancy size, AFM-YOLOv8s’ 6.87 (×10^6^) parameter size and 13.4 (MB) model size were both the least among the 7 models. In terms of FPS and detection time per image, YOLOv3-tiny obtained the highest FPS and the least detection time, followed by YOLOv8s. AFM-YOLOv8s experienced a slight decrease in speed compared to the benchmark model, with values of 71.2 (frames/s) and 0.011 (image/s), respectively.

**Table 8.** Comparisons with state-of-the-art detection methods with metrics of mAP50 (Eq. 7), F1 score (Eq. 8), FPS, parameter size, detection time, and model size. Note that bold font indicated the optimal result.

Models	Backbone	mAP50	F1	Parameter size (×10^6^)	Model size (MB)	FPS (frames/s)	Detection time (image/s)
Faster R-CNN	ResNet50	0.874	0.727	41.4	315.1	21.5	0.082
RetinaNet	ResNet50	0.845	0.699	36.4	245.5	20.4	0.085
RT-DETR	ResNet50	0.851	0.842	42.0	86.1	24.6	0.041
YOLOv3-tiny	Darknet53	0.876	0.827	12.13	23.2	**109.9**	**0.003**
YOLOv5s	CSPDarknet53	0.885	0.829	9.11	17.6	75.8	0.008
YOLOv8s	CSPDarknet53	0.886	0.832	11.16	21.5	106.5	0.006
AFM-YOLOv8s	CSPDarknet53	**0.913**	**0.853**	**6.87**	**13.4**	75.6	0.011

**Fig. 9.**
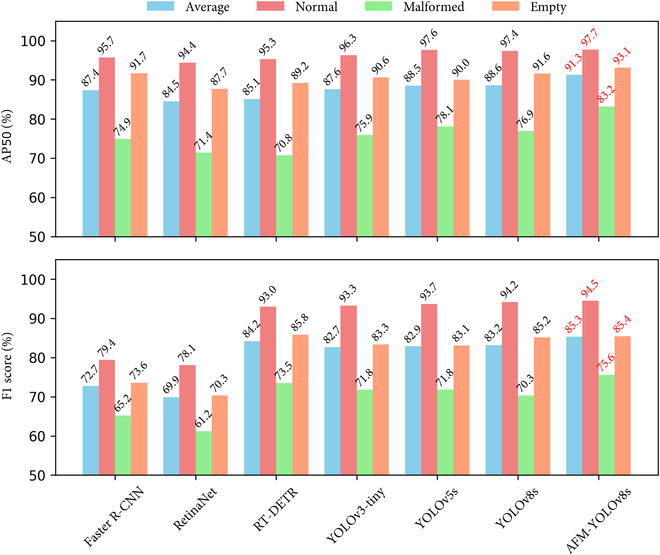
Model comparison for the detection of multiform sporangia categories using AP50 and F1 score metrics. AP50 is the performance evaluation metric that refers to the average precision at an IoU threshold of 0.5 for each sporangia category.

Figure 9 shows the average precision and F1 score of different models on 3 morphological variant sporangia categories. Among the different models, the AFM-YOLOv8s achieved the highest average precision of 91.3%, as well as high accuracy in each category: normal at 97.7%, malformed at 83.2%, and empty at 93.1%. In addition, in terms of F1 score, AFM-YOLOv8s also obtained the highest scores, with normal at 94.5%, malformed at 75.6%, and empty at 85.4% across the 3 sporangia categories.

**Fig. 10.**
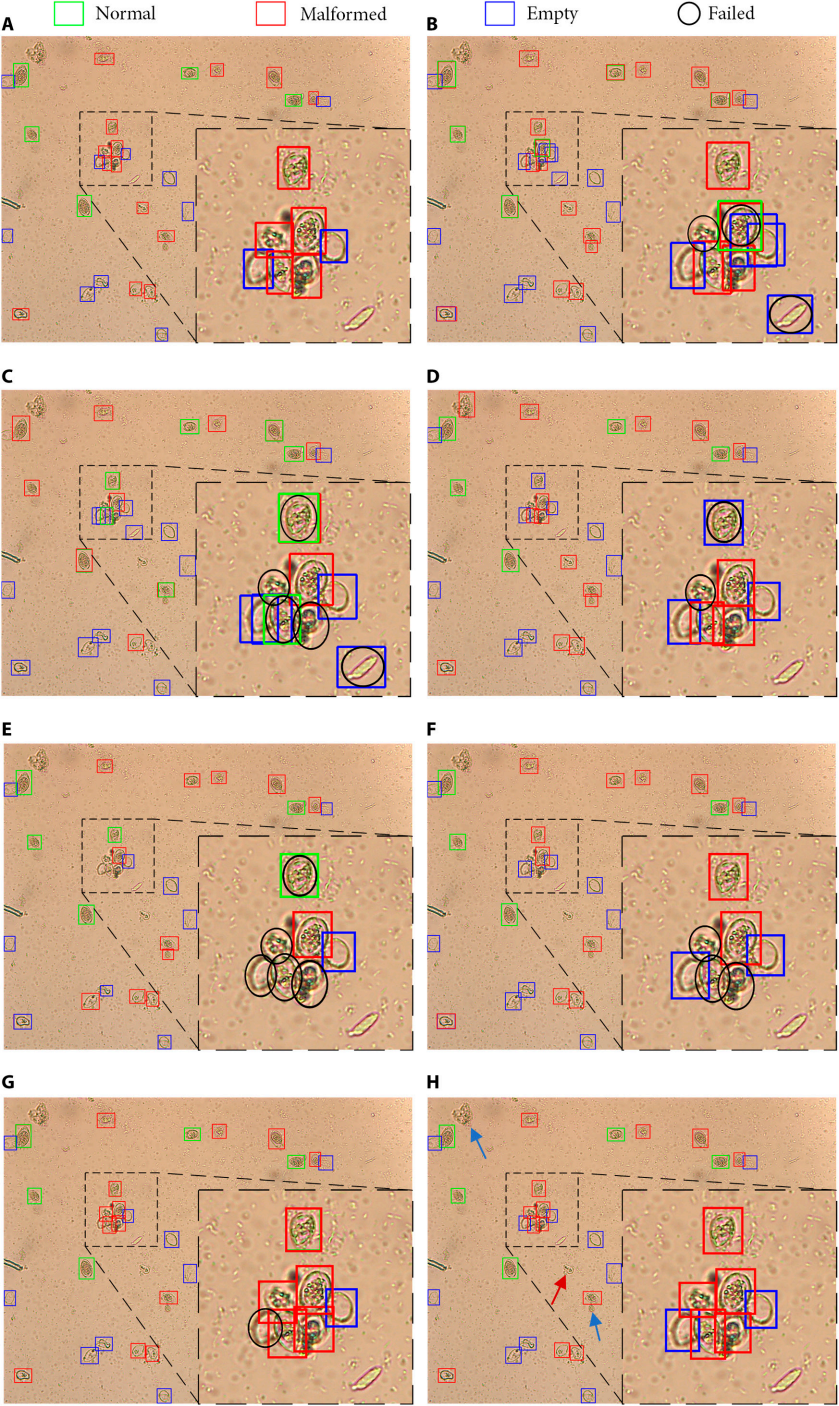
Examples of detection results of sporangia with high density and complex background by 7 models (B to H). The orange background is naturally from the microscope. (A) Original image with ground-truth bounding box, (B) Faster R-CNN, (C) RetinaNet, (D) RT-DETR, (E) YOLOv3-tiny, (F) YOLOv5s, (G) YOLOv8s, and (H) AFM-YOLOv8s.

The visualization of detection results is shown in Fig. 10, where the black dashed boxes indicate zooming in on the target area. Faster R-CNN, RT-DETR, YOLOv8s, and AFM-YOLOv8s had better detection results when sporangia show densification, occlusion, and the presence of the effect of background noise (Fig. 10). These models could detect the majority of the sporangia in the target area. Only AFM-YOLOv8s was able to detect all of the grapevine downy mildew sporangia and correctly classify them, while other models missed a certain number of sporangia due to reasons like blurred backgrounds or special location (Fig. 10). Despite its superior performance over other models, AFM-YOLOv8s still misses 3 sporangia, as indicated by the blue and red arrows in Fig. 10H. The blue arrows highlight unmarked sporangia that are challenging for annotators to distinguish, while the red arrow points to missed sporangia that were marked.

The other detection results are shown in Figs. S4 and S5. In Fig. S4, the black dashed boxes indicate a zoomed-in view of the target area. There are 9 sporangia in this area as shown in Fig. S4A, where red arrows point to sporangia undergoing occlusions and blue arrows indicate sporangia undergoing substantial morphological changes. In the zooming area with 9 sporangia, RT-DETR, YOLOv3-tiny, YOLOv5, and AFM-YOLOv8s demonstrated relatively high detection accuracy, detecting more than 6 sporangia successfully. In particular, AFM-YOLOv8s stood out by accurately detecting and classifying all sporangia, whereas other models faced difficulties when dealing with occlusions and substantial morphological changes sporangia (Fig. S4). In Fig. S5, red arrows indicate 6 sporangia that are occluded because of being on the edge of the image or obscured by other sporangia. For these occluded sporangia, RT-DETR, YOLOv3-tiny, YOLOv5s, and YOLOv8s detected most of them with some missing cases, while only AFM-YOLOv8s detected all of them (Fig. S5).

## Discussion

Detecting fungal sporangia across various forms amidst complex backgrounds poses a considerable challenge yet is crucial for monitoring disease epidemiology and studying fungicide resistance. The AFM-YOLOv8s model, enhanced with 3 innovative modules, demonstrates superior accuracy, efficiency, and robustness in the detection and classification of 3 types of sporangia compared to established algorithms like YOLOv8s. To facilitate practical applications, a web application utilizing AFM-YOLOv8s was developed, enabling automatic sporangia detection and counting. This tool significantly accelerates the speed and reduces labor in disease monitoring and resistance studies, streamlining critical processes in plant pathology.

### FasterCSP: Trade-offs between accuracy and speed

For a given platform and application in object detection, trade-offs are observed between speed, memory, and accuracy, and a certain balance needs to be achieved [36,37]. In this study, the integration of FasterCSP, which replaced the C2f modules in both the backbone and neck, resulted in a slight decrease in performance but gained a significant reduction in the model’s complexity, as indicated by decreases in the number of parameters (by 1.76 × 10^6^), GFLOPs (by 6.1), and model size (by 3.4 MB). The model size reduction makes it possible to deploy the model to hardware with limited computational capacity, such as small phones or embedded devices, without compromising on its detection capabilities [38,39]. This increased versatility opens up opportunities for widespread use in real-world applications where resource constraints are a consideration. Moreover, the lighter model also facilitates faster inference times, enabling quicker real-time detection of objects, which is particularly beneficial for use cases like automatic spore traps [5]. In addition, the reduced model size makes it easier to distribute and update the model over networks, ensuring seamless integration into existing systems and workflows. Therefore, we chose FasterCSP over C2f and compensated the accuracy loss by following information integration model, ACF-FPN.

### The effects of multiscale feature information integration with ACF-FPN

Detecting sporangia in various forms against complex backgrounds poses a significant challenge, even for many of the state-of-the-art models [7,40]. Embedding ACF-FPN to YOLOv8s notably boosted the detection accuracy, 2.3% in mAP50 and 3.0% in F1 scores. This outcome can be attributed to the ACF-FPN’s advantage in fusing multiscale feature information extracted from the backbone network, underscoring the importance of utilizing multiscale feature information for detection of small targets like various forms of sporangia of grapevine downy mildew. Similar efforts have been made by other studies. For instance, the BiFPN applies top-down and bottom-up path fusion of multiscale features iteratively [29]. Asymptotic FPN (AFPN) iteratively fuses high-level and low-level features through a progressive FPN [27], while generalized FPN (GFPN) fully utilizes feature information from different dimensions through cross connections and skip connections [28]. All these modules achieved significantly improved model accuracy (mAP50 increases by more than 1.3% and F1 scores increase by more than 1.2%) over the original PANet feature fusion module of the YOLOv8, further emphasizing the importance of multiscale feature information utilization. Compared to these models, ACF-FPN achieved a higher accuracy regarding mAP50 and F1 scores. The reason can be attributed to advanced fusion methods adopted by ACF-FPN. Simple bidirectional connections in BiFPN might lead to insufficient information fusion. While AFPN achieves comprehensive fusion of multiscale feature information with a progressive structure, it overlooks potential information loss during the information transfer process. GFPN, despite reducing information loss with skip connections, does not consider the varying contributions of different features to recognition. Accordingly, ACF-FPN introduced bidirectional connections, which ensured the comprehensive interactions between low-level spatial information and high-level semantic information. This approach reduced semantic gaps between different dimensions through adaptive fusion. In addition, to avoid potential information loss and gradient loss during multilevel transmission, ACF-FPN added residual connection from initial feature input to final output. Moreover, ACF-FPN used the CBS module to unify the number of channels, thereby reducing the overall parameter count and computation while enhancing accuracy and maintaining a lightweight structure.

### MPDIoU: A better loss function for locating bounding boxes

The loss function, a critical component in object detection model development, quantifies the disparity between predicted output and ground-truth annotations. MPDIoU, a bounding box similarity metric, integrates essential factors such as intersection ratio, center point distance, and width and height deviation by leveraging the minimum point distance [26]. The adoption of MPDIoU enhanced AFM-YOLOv8s ability in locating the bounding box and learning the spatial localization of objects within an image (Table 5 and Fig. S3). The obviously improved performance demonstrates that specific modules refinements to the model can effectively address common performance bottlenecks, including performance degradation on complex datasets, inaccuracies in bounding box position predictions, and occurrences of false positives and false negatives during the detection process. Furthermore, similar improvements have been observed in other studies, such as [41,42], indicating the broader applicability and effectiveness of incorporating MPDIoU into detection models. These findings underscore the importance of refining specific model modules to effectively address common performance challenges in object detection.

### ACF-FPN’s better compatibility with YOLO than ResNet50-based models

The YOLO model family, comprising YOLOv3, YOLOv5, and YOLOv8, consistently outperforms ResNet50-based models such as Faster R-CNN, RetinaNet, and RT-DETR in various tasks. Notably, AFM-YOLOv8s exhibits superior classification performance compared to all other methods for multitarget classification tasks within complex datasets (Fig. 9). This difference in performance could stem from the distinct backbones adopted by YOLO models and ResNet50-based models. YOLOv3 and other YOLO variants utilize larger feature extractors like Darknet53 and improved CSPDarknet53, comprising 53 convolutional layers with residual connections [43], which notably enhance feature extraction capability compared to ResNet50 used in other models. The discovery that the Darknet53 backbone achieves top 1 and top 5 accuracy levels comparable to ResNet152 in classification tasks [44] aligns with our results. Hence, with the different power for feature extraction, the different backbone could affect the accuracy of corresponding models.

For the detection of 3 types of sporangia—normal, empty, and malformed —all methods demonstrated significantly higher accuracy in detecting normal and empty sporangia compared to malformed ones. This disparity may be attributed to the abundance of samples available for normal and empty sporangia, whereas there might be insufficient samples for malformed ones. In addition, the relatively consistent morphological features of normal sporangia contribute to their high-accuracy detection, while the variability in morphology of malformed sporangia, characterized by substantial differences at different time intervals during the entire chemical substance release process, may lead to decreased accuracy. Thus, it can be inferred that both the polymorphism of sporangia and the number of samples can significantly impact model accuracy [45–47].

In summary, both backbones adopted and the property of datasets could impact the performance of target detection. This highlights the potential application value of improving the model based on dataset characteristics to enhance multicategory detection.

### AFM-YOLOv8s for detecting sporangia under complex conditions

Object detection under complex backgrounds is challenging because of shadows, similar textures, visual clutter, and occlusion, which make it difficult to distinguish objects from their surroundings [48]. AFM-YOLOv8s proved its superior performance in detecting sporangia under complex conditions (Fig. 10 and Figs. S4 and S5). Compared with other models derived from YOLOv8, AFM-YOLOv8s uses an improved feature fusion and bounding-box-based loss function, which yields an improved accuracy and a high robustness [17,18]. Feature fusion integrates multilevel features from different layers of the used neural network, allowing AFM-YOLOv8s to capture both high-level semantic information and details at once [49]. This combination enhances the model’s ability to recognize sporangia in complex backgrounds by providing a more comprehensive understanding of the context and spatial relationships between objects [50]. Meanwhile, the MPDIoU loss function directly optimizes the prediction of bounding boxes, ensuring precise localization of the sporangia, even with occlusions [51]. By punishing discrepancies between predicted and ground-truth bounding boxes, this loss function incentivizes the model to generate accurate bounding box coordinates, improving both localization accuracy and overall object detection performance. Together, these improvements mitigate common challenges like occlusion, scale variation, and background clutter, leading to more reliable and robust object detection results.

### Potential usage in disease monitoring and management

Pathogen spore monitoring is of paramount importance in early disease detection and forecasting since it can optimize the timing of preventive crop protection measures [52]. It enables targeted interventions only when and where they are needed [53]. It facilitates a more objective evaluation of fungicide efficacy and also allows for the early detection of changes in pathogen populations, including the emergence of fungicide-resistant strains [54,55]. This will ultimately provide guidance for the research and development of fungicides and their scientific and rational spatiotemporal layout, so as to maximize the efficacy of fungicides, extend the life cycle of products, and effectively reduce or delay the emergence of drug-resistant strains. Although numerous studies have explored the use of target detection algorithms for spore detection [6,7,56,57], these efforts mostly remain in the theoretical research phase with limited practical applications due to limitations in accuracy and speed. AFM-YOLOv8s achieves its practicality for real-time detection needs and can be extended to other types of spores like rust, fungal, and bacterial. Leveraging the high-performance object detection capability of AFM-YOLOv8s, we developed an automated sporangium detection web application for grapevine downy mildew to replace traditional-labor-intensive manual sporangium detection processes by providing fast and accurate results with the input of microscope images into the model. Furthermore, we provide this application with a user-friendly interface that is easy to scale (http://118.89.50.72/detect). We believe that assimilating sporangia count data into disease models, allowing for a more accurate representation of disease dynamics and more reliable disease intervention measures.

### Limitations

Despite the aforementioned merits, AFM-YOLOv8s has 2 main limitations. First, regarding algorithm classification accuracy, as evident from the experimental results in Table 8 and Fig. 9, although AFM-YOLOv8s achieved high accuracy (>90%) in recognizing normal sporangia and empty sporangia, it fell short of expectations in identifying malformed sporangia, with an accuracy of 83.2%, which resulted in a decrease in the overall average accuracy. The primary reason for this discrepancy may be the imbalance in the data. Malformed sporangia induced by chemical treatment constitute a much smaller proportion in sporangia micrographs, leading to a lower instance ratio in the dataset (<20%) compared to other categories. Despite the use of distribution focal loss function in YOLOv8 algorithm to assign higher weights to imbalanced classes during classification loss computation [58], the detection accuracy is still relatively low with respect to the normal and empty sporangia. Moreover, despite achieving an overall accuracy of 91.3% and the presence of manual tagging errors, missed sporangia instances can still occur, as indicated by arrows in Fig. 10H. Second, in terms of the dataset, although diverse strains were used in constructing the dataset, given the complexity and diversity of microbial populations in natural fields, the current dataset could be further enriched.

To conclude, the development of the AFM-YOLOv8s model achieved a significant advancement in the detection and classification of fungal sporangia of various forms amidst complex backgrounds. Through its superior accuracy, efficiency, and robustness, AFM-YOLOv8s offers practical solutions for disease monitoring and resistance studies. Furthermore, the integration of FasterCSP, ACF-FPN, and MPDIoU into the model demonstrates the potential for achieving a balance between speed, memory, and accuracy, enhancing its versatility and real-world applicability. The use of AFM-YOLOv8s in developing an automated sporangia detection web application underscores its practical utility in replacing labor-intensive manual detection processes. Moving forward, the assimilation of sporangia monitoring data into disease models holds promise for improving disease forecasting and intervention strategies, emphasizing the importance of continued advancements in detection technology for effective disease management.

## Data Availability

The data and code used in this study are available on GitHub (https://github.com/Lzzyyy123/spore_detect). The URL of web application of sporangia detection is http://118.89.50.72/detect.
