# Supplementary material for: AFM-YOLOv8s: An Accurate, Fast, and Highly Robust Model for Detection of Sporangia of Plasmopara viticola with Various Morphological Variants
Source: Plant Phenomics. 2024 Sep 11;6:0246. doi: 10.34133/plantphenomics.0246 (PMC11387751; doi:10.34133/plantphenomics.0246)
Supplement: Supplementary 1 — Figs. S1 to S5 [file plantphenomics.0246.f1.docx]

# Supplementary Materials


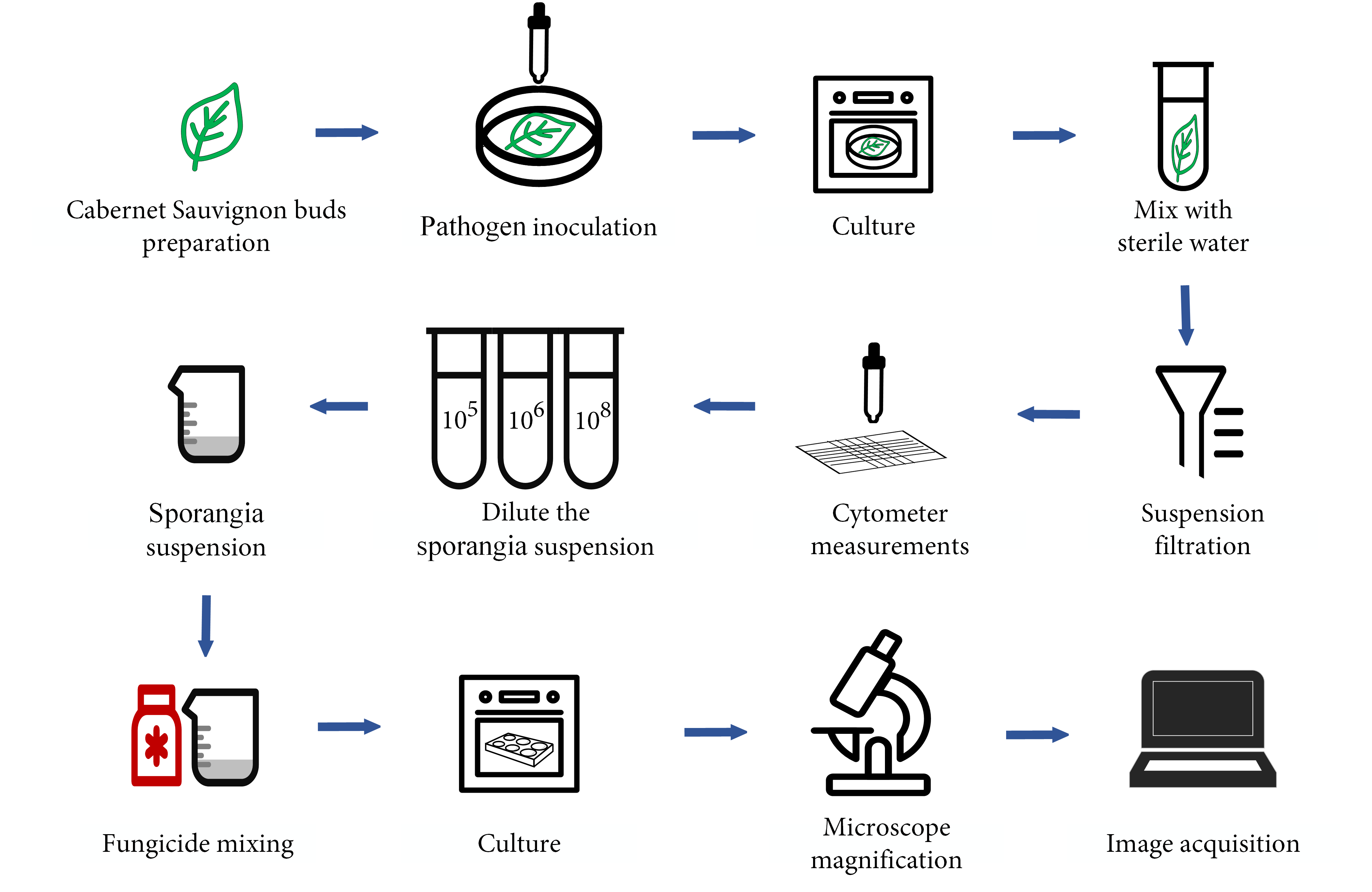


**Fig. S1.** Workflow for sporangia extraction and microscopic image acquisition.


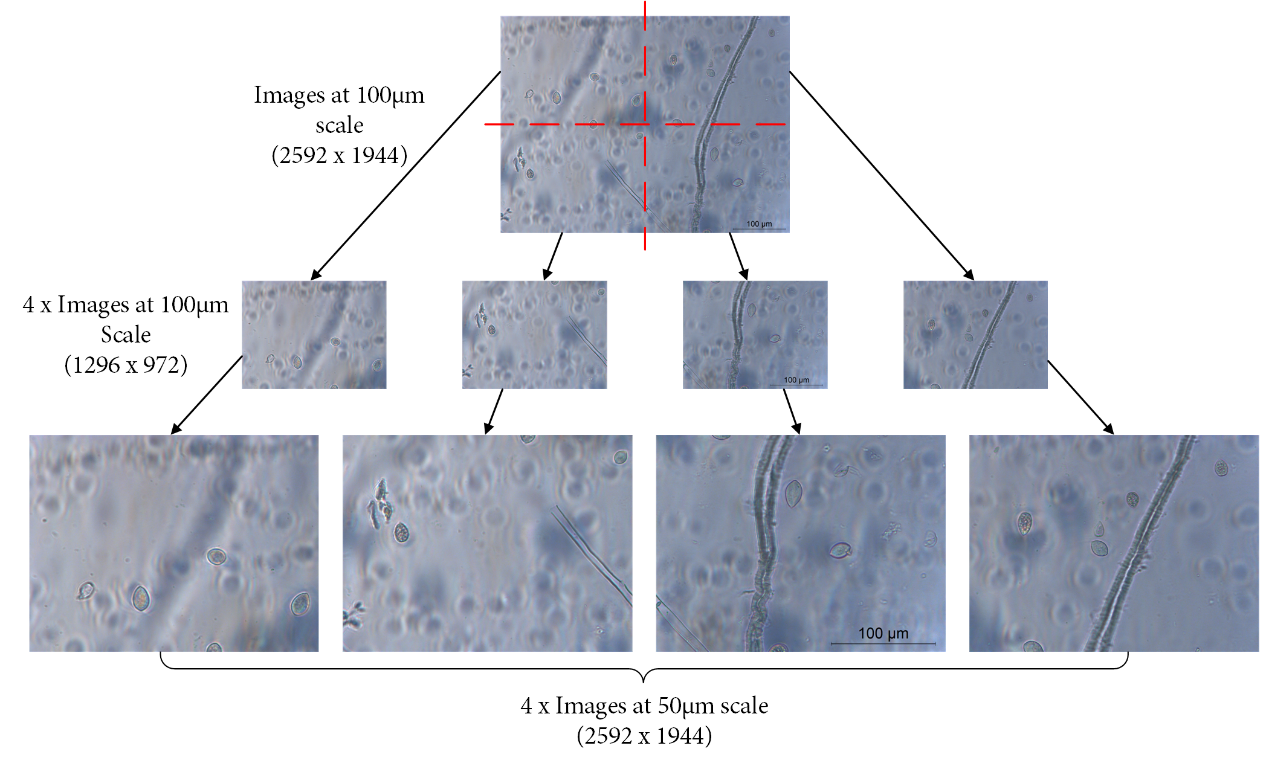


**Fig. S2.** Workflow for multi-scale comparison experiments image generation.


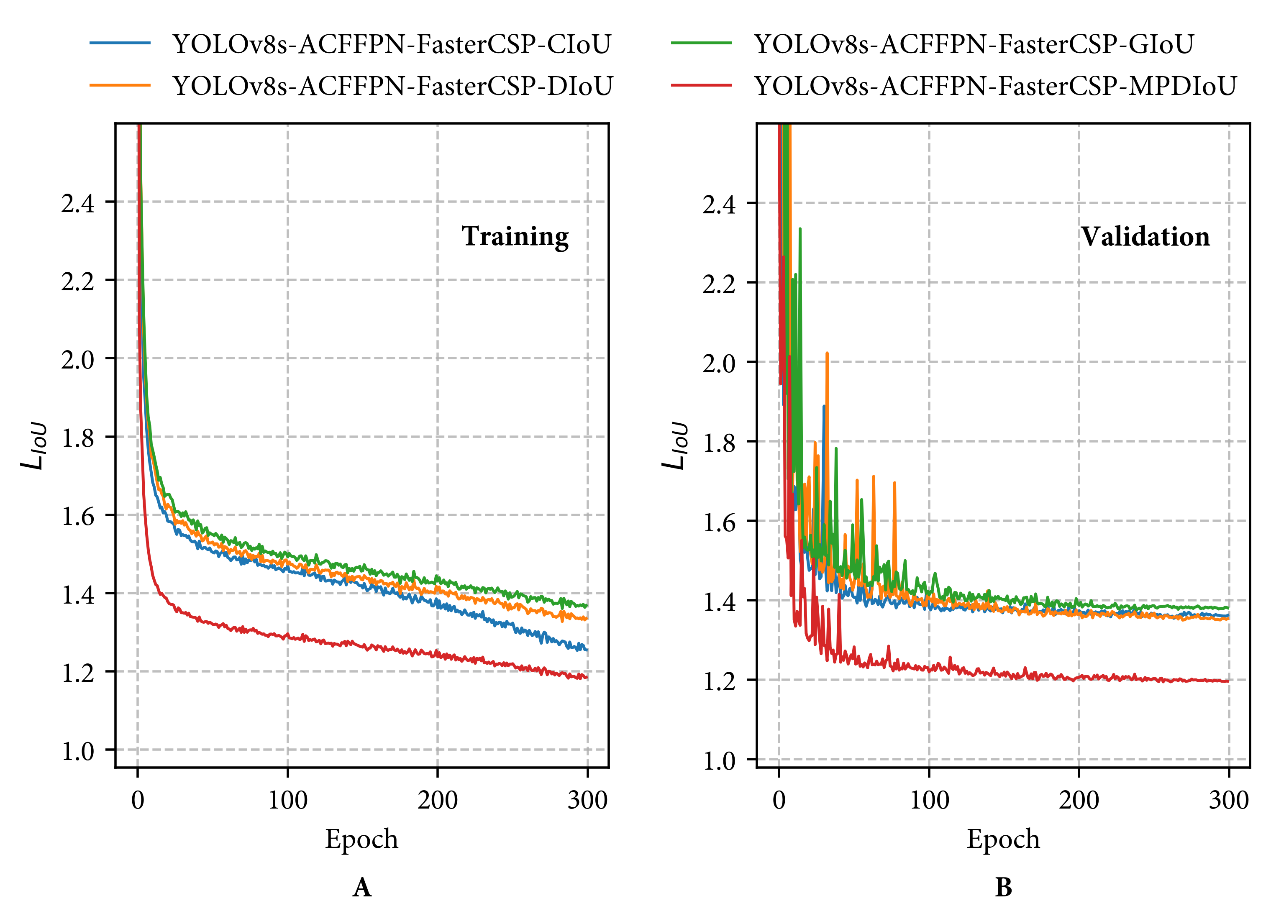


**Fig. S3.** Comparisons of the effect of different bounding box loss function on model performance at training (A) and validation (B) phases.


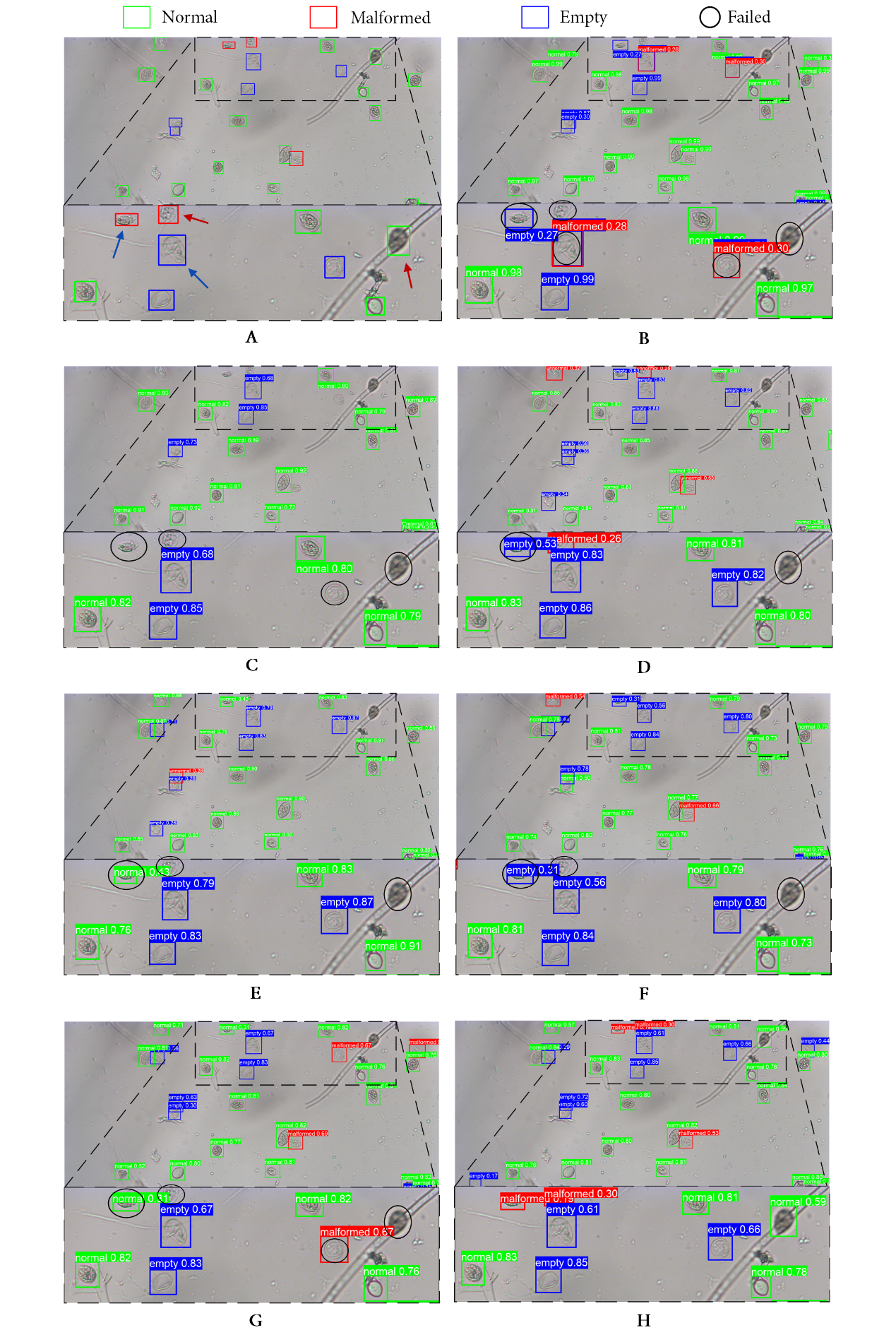


**Fig. S4.** Examples of detection results for sporangia with occlusions and significant morphological changes by seven models (B-H). The gray background is naturally from the microscope. (A) Original image with ground truth bounding box (B) Faster R-CNN (C) RetinaNet (D) RT-DETR (E) YOLOv3-tiny (F) YOLOv5s (G) YOLOv8s and (H) AFM-YOLOv8s.


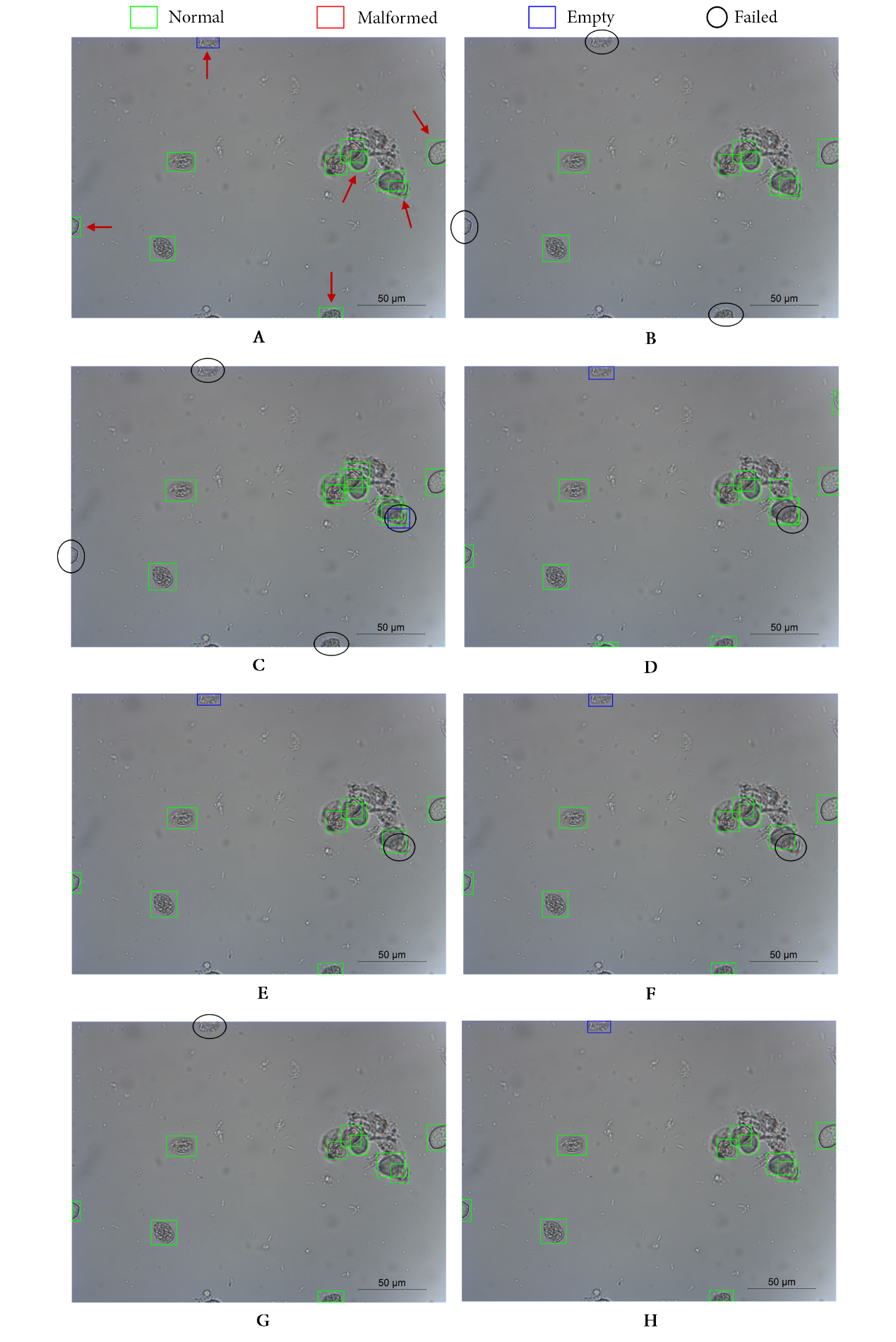


**Fig. S5.** Examples of detection results of sporangia with occlusion by seven models (B-H). The gray background is naturally from the microscope. (A) Original image with ground truth bounding box (B) Faster R-CNN (C) RetinaNet (D) RT-DETR (E) YOLOv3-tiny (F) YOLOv5s (G) YOLOv8s and (H) AFM-YOLOv8s.
